# HearteXplain: explainable prediction of acute heart failure and identification of hematologic biomarkers using EBMs and Morris sensitivity analysis

**DOI:** 10.1038/s41598-025-23668-7

**Published:** 2025-11-14

**Authors:** Fatma Hilal Yagin, Yasin Görmez, Abdulmohsen Algarni, Fahaid Al-Hashem, Ashit Kumar Dutta, Mohammadreza Aghaei

**Affiliations:** 1 Department of Biostatistics, Faculty of Medicine, Malatya Turgut Ozal University, Malatya, 44210 Turkey; 2 Department of Management Information Systems, Faculty of Economics and Administrative Sciences, Sivas Cumhuriyet University, Sivas, 58140 Turkey; 3 Department of Computer Science, King Khalid University, Abha, 61421 Saudi Arabia; 4 Department of Physiology, College of Medicine, King Khalid University, Abha, 61421 Saudi Arabia; 5 Department of Computer Science and Information Systems, College of Applied Sciences, AlMaarefa University, Kingdom of Saudi Arabia, Ad Diriyah, Riyadh, 13713 Saudi Arabia; 6 Department of Ocean Operations and Civil Engineering, Norwegian University of Science and Technology (NTNU), Alesund, Norway; 7 Department of Computer Science, Lakehead University, Thunder Bay, ON P7B 5E1, Canada; 8 Research Center, Deanship of Scientific Research and Post-Graduate Studies, AlMaarefa University, Dariyah, 13713, Saudi Arabia; 9 Department of Sustainable Systems Engineering (INATECH), Albert Ludwigs University of Freiburg, Freiburg, Germany

**Keywords:** Acute heart failure, Explainable artificial intelligence, Biomarkers, Morris sensitivity analysis, Diagnostic modeling, Model interpretability, Bayesian optimization, Biomarkers, Computational biology and bioinformatics, Medical research

## Abstract

Hematological biomarkers have emerged as powerful tools in diagnosing Acute Heart Failure (AHF). This study introduces a novel diagnostic framework that integrates Explainable Artificial Intelligence (XAI) with Morris Sensitivity Analysis (MSA) to enhance both the interpretability and performance of machine learning models in AHF detection. A dataset consisting of 425 AHF patients and 430 controls was analyzed using eight machine learning models, including XGBoost, Histogram-based Gradient Boosting (histGB), Explainable Boosting Machine (EBM), and Random Forest. Model performance was evaluated through metrics such as AUC, accuracy, precision, recall, and Brier score. Hyperparameters were optimized via Bayesian optimization. Feature importance was assessed using MSA to identify variables with the highest predictive influence. The histGB model achieved the highest performance with an AUC of 87.93%. Both MSA and EBM consistently identified PDW, RDW-CV, NEU, NEU/LY ratio, age, and WBC as top predictive features across multiple models. These hematological markers demonstrated strong potential for early diagnosis and risk stratification in AHF patients. This study presents a clinically relevant, interpretable, and cost-effective diagnostic strategy that combines XAI with MSA for AHF prediction. The framework enhances clinical trust and provides a pathway toward personalized treatment by identifying accessible hematological biomarkers. The integration of explainability into AI models improves their transparency and applicability in real-world clinical settings.

## Introduction

Acute heart failure (AHF) is defined by the sudden appearance or worsening of clinical symptoms and signs of heart failure, which are the primary causes of unscheduled hospital admission and emergency treatment^1,2^. AHF, primarily caused by a sudden deterioration of chronic heart failure, necessitates hospitalization and has the potential to be fatal. Therefore, it is crucial to promptly diagnose and initiate therapy in patients who arrive at the emergency room. Previous research has shown that individuals with AHF tended to be rehospitalized within a period of 30 to 90 days after being discharged, making up approximately 25 to 30% of all patients. The unplanned short-term readmission of patients with AHF not only imposes a significant financial burden on the national healthcare system but also leads to severe harm or significantly impacts the patient’s quality of life^3,4^. AHF is hard to diagnose without using a lot of different clinical tests and biomarkers, like brain natriuretic peptide (BNP) and N-terminal proBNP (NT-proBNP), as well as electrocardiography (ECG), echocardiogram (ECHO), and blood tests. However, other conditions like end-stage heart failure and chronic renal disease can influence these markers, thereby complicating the diagnosis process. Hematologic markers have been explored as one of the additional techniques to enhance the clinical evaluation of patients at risk for cardiovascular illnesses^5–7^. The relationship between hematological biomarkers and different outcomes has been investigated in many studies^8,9^. These biomarkers, which may be found in a common and affordable blood count test, can be valuable for more accurate evaluation of patients and identification of individuals with a high risk of mortality. Multiple studies have demonstrated a correlation between elevated red blood cell distribution width (RDW) and unfavorable prognosis in individuals suffering from cardiac disease^10–13^. Additional research has demonstrated that an elevated neutrophil-lymphocyte ratio (NLR) and platelet-lymphocyte ratio (PLR) can be used to predict the occurrence of heart failure^14–18^. Furthermore, a greater PLR^19,20^ is linked to unfavorable outcomes.

In recent years, Artificial Intelligence (AI) has attracted prominence in systems used for clinical decision-making, offering significant potential to emulate human reasoning and analyze intricate medical data. Explainable AI (XAI) improves this capability by providing transparency and interpretability, thereby allowing healthcare professionals to make well-informed decisions based on the recommendations made by the AI system. The objective of this study is to combine XAI with hematological markers to create a new diagnostic method for AHF. The ultimate goal is to enhance clinical practices and improve patient outcomes. Sensitivity analysis (SA) techniques serve a crucial role in enhancing our comprehension of the influence and unpredictability of characteristics or variables in machine learning (ML) models, simulators, and real-world applications. SA offers valuable insights into machine learning models and applications, making it a model-independent method for XAI. XAI is becoming more crucial in the field of AI research and applications. In many instances, just providing inference is insufficient. Domain experts and engineers seek to address many key problems before using AI approaches in production, including understanding the inner workings of an ML model, identifying the factors that influence predictions, and assessing the uncertainties inherent in both the data and the model. SA is crucial in the area of XAI since it may provide answers to these problems independent of any particular ML model or sampling technique. SA is used in several practical applications, such as comprehending the functioning of chemical models^21^ and measuring the impact of input parameters on biological models^22^. Morris SA (MSA) involves multidimensional averaging (i.e., assessing the influence of one factor while others vary) and is an approach that allows for the grouping of factors, making it particularly suitable for working with very large datasets. In addition, the modified version of the MSA, which takes into account the absolute means of the distribution of main effects, is robust in terms of type II errors^23^. There are numerous studies in the literature applying classical ML approaches for AHF detection, but no study has developed a prediction model and biomarker discovery using MSA-based XAI approaches in AHF patients. This research provides new insights and methodologies for early and accurate diagnosis of AHF by analyzing hematological markers through MSA-based XAI. We selected hematological markers because they are routinely collected, inexpensive, and provide rapid diagnostic information compared with more costly biomarkers such as BNP or NT-proBNP. These variables have been consistently linked with prognosis in heart failure, yet their diagnostic utility in AHF remains underexplored. We employed MSA for feature selection due to its model-agnostic nature, ability to capture both main and interaction effects, and robustness in ranking features even in high-dimensional spaces. This ensures that selected predictors are both statistically influential and clinically interpretable.

The novelty of this work lies in the integration of Explainable Boosting Machines (EBMs) with MSA to identify hematological biomarkers for AHF. Unlike previous studies that either applied black-box machine learning models or focused solely on statistical associations, our framework uniquely combines model interpretability with systematic sensitivity analysis. This dual approach not only improves predictive performance but also provides mechanistic insights into biomarker contributions. Additionally, the methodology was validated on a relatively large and homogeneous dataset with repeated holdout resampling, ensuring robustness of results. To the best of our knowledge, no prior study has employed MSA in conjunction with EBMs to discover and validate hematological predictors of AHF.

## Materials and methods

### Data collection and characteristics

This study used retrospectively available data of patients admitted to the hospital with AHF. All participants were recruited from Turkey, and the cohort is ethnically homogeneous. The study used the following inclusion criteria: 425 patients aged 18 years and older who presented with signs or symptoms of fluid retention, including dyspnea, paroxysmal nocturnal dyspnea, orthopnea, ankle edema, or jugular venous distension due to worsening heart failure were accepted. Additionally, a control group of 430 healthy individuals without HF who presented to the cardiology department with nonspecific cardiac complaints such as weakness, fatigue, and palpitations underwent normal cardiac examination and echocardiography. The following exclusion criteria were used in the study: patients with hematologic malignancies, those taking medications that affect the complete blood count (e.g., chemotherapy drugs that suppress the bone marrow or overdose of warfarin), those with hemoglobin less than 10 g/dL, those with active bleeding, those with acute infections, those with acute myocardial infarction, those with severe renal impairment (eGFR less than 15 mL/min/1.73 m^2^), those with severe hepatic impairment, those with chronic obstructive pulmonary disease, and those with chronic inflammatory diseases (during acute exacerbation of disease)^24^. We performed an independent samples t-test with the G*Power program (University of Düsseldorf, Düsseldorf, Germany, version 3.0.1) to determine the sample size and actual power. The parameters used were α = 0.05, power = 0.80, and effect size = 0.2. The findings indicated that the true power was 80%, requiring a minimum sample size of 394 people in each group. We conducted the study by the Declaration of Helsinki and received approval from the Inonu University Non-Interventional Research Ethics Committee (decision number: 2024/6190). After all exclusion procedures, the dataset used for the analysis of machine learning models comprised a total of 855 instances, of which 369 (43.2%) were from female participants and 486 (56.8%) from male participants. The sex variable was also included as a feature in the models to enable the analysis of potential differences in disease patterns between female and male participants.

### Model development and performance evaluation

This study used eight machine learning models to predict AHF: Explainable Boosting Machine (EBM), Extra Trees Classifier (ETC), Histogram-based Gradient Boosting (histGB), KTBoost, Logistic Regression (LR), Multi-Layer Perceptron (MLP), Random Forest (RF), and XGBoost. We used the repeated holdout method for hyper-parameter optimization, training, and testing the models. The Bayesian optimization technique optimized the hyper-parameters of the models for each holdout. We computed the model performances using performance metrics, including accuracy, precision, recall, area under the curve (AUC), Brier score, and decision curve analysis. Finally, we explained the models using MSA^25^.

### Classification model

EBM is a machine learning method based on generalized additive models that balances transparency and performance. EBM uses ensemble methods, such as decision trees, to clearly show the contribution of each feature. EBM can easily detect complex relationships and nonlinear interactions between features, thanks to its ability to capture monotonicity constraints and interaction effects. While EBM enhances model performance, it also aims to convey the model’s internal workings to the user^26^.

ETC is a variation of the random forest algorithm, and its main purpose is to classify using decision trees. This method creates many decision trees and trains each tree on random subsets of the training data and its features. The decision trees randomly choose the split points, unlike the random forest algorithm. The algorithm selects a completely random point instead of searching for the best-split point. Thanks to randomness, ETC aims to prevent overfitting while increasing model training speed^27^.

The histGB is an optimized version of the classic gradient boosting algorithm. The histGB method separates continuous features into histograms, thereby reducing the data into fewer bins. Histograms create features in the data set by dividing them into specific intervals, thereby reducing computational cost and memory usage. The histGB works faster and more efficiently on large datasets and high-dimensional feature spaces. The histGB strives to reduce model training time and produce high-accuracy predictions without sacrificing performance. Additionally, the histGB allows large datasets to fit in memory and perform faster operations with parallel calculations^28^.

KTBoost is a strategy that combines kernel approaches with the gradient boosting algorithm. KTBoost simplifies the process of modeling intricate and non-linear patterns by using kernel functions to capture the connections between data points. KTBoost utilizes the flexibility of kernel functions to effectively discover the hidden structures in the data space. Kernel functions map data points into a higher-dimensional space, facilitating their separation. KTBoost seeks to enhance the accuracy and generalizability of models by incorporating these projections into the gradient boosting framework and minimizing the error at each iteration^29^.

LR is a widely used simple statistical method in classification problems, particularly effective in binary classification problems. LR uses the sigmoid function, which produces values between 0 and 1 from predicted probabilities, to model the relationship between the independent variables and the dependent variable. The model generates the logit function by establishing a linear relationship with a transformation known as log-odds. In addition to all these, it uses regularization techniques such as L1 and L2 to prevent overlearning^30^.

An MLP is a fundamental kind of artificial neural network that has a minimum of three layers: input, hidden, and output. It serves as a foundational component of deep learning. The process involves each layer receiving inputs from the preceding layer, multiplying them by weights, applying an activation function, and forwarding them to the subsequent layer. The use of commonly employed activation functions such as sigmoid, tanh, and ReLU helps the neural network in acquiring knowledge about non-linear connections. During the learning phase, an MLP carries out classification as a feed-forward network and adjusts its weights using the error-back propagation technique. Our approach involves using both feed-forward and feedback mechanisms to reduce the discrepancy between the anticipated and observed outputs^31^.

We build RF, a powerful ensemble method, by combining many decision trees. This method assembles numerous decision trees and trains each tree on random subsets of the dataset. In the final stage, RF aims to enhance the model’s generalization capacity by making its final prediction based on the majority vote or average of the trees. Parallel computing efficiently trains RF, reducing the risk of overfitting to provide more robust and reliable predictions, enabling it to produce results quickly on large data sets^32^.

XGBoost is a refined iteration of the gradient boosting technique that provides superior precision and effectiveness. XGBoost employs decision trees as weak learners, with each subsequent tree aiming to rectify the mistakes made by the preceding trees. XGBoost seeks to enhance the performance of a model by generating more precise error estimates via the use of quadratic derivatives. An outstanding characteristic of the XGBoost approach is its ability to effectively handle huge datasets via its parallel computing and distributed processing capabilities, resulting in fast performance. Additionally, it employs regularization approaches to mitigate the issue of overfitting. The tree structure is made more compact and efficient by using a feature called “sparse awareness”, which allows it to properly manage missing data^33^.

### Validation method

For both model assessment and validation, we use the repeated holdout method, a cross-validation approach. The repeated holdout approach is a variation of the basic holdout method that assesses performance by dividing the dataset into separate training and testing groups. The repeated holdout technique seeks to assess the model’s performance in a more reliable and statistically sound manner by repeating this procedure numerous times. In each iteration, we use a random partitioning technique to split the dataset into separate training and test sets. Subsequently, we proceed to train the model using the training set and assess its performance using the test set. A performance metric is computed for each iteration of this method, which is repeated a certain number of times. We selected repeated holdout cross-validation instead of a single train–test split because it provides a more reliable estimate of model performance by reducing the dependency on a single random partition of the dataset. This approach mitigates the variance inherent in single splits and yields statistically robust results by averaging across multiple iterations. By doing so, we are able to correctly depict the overall performance of the model by calculating the average performance metrics from all of the replications^34^. In this study, the repeated holdout procedure was performed 100 times, and the final results were averaged across these iterations to ensure stability and reduce random variation.

### Bayesian optimization

Particularly for optimizing costly and intricate functions, we use the Bayesian optimization approach. High computational cost optimization applications, like hyperparameter tuning, frequently employ Bayesian optimization. Bayesian optimization utilizes a prior assumption to make predictions about the performance of a model and then updates this belief with fresh evidence. Typically, Gaussian processes begin the optimization process by estimating the unknown distribution of the goal function. Subsequently, we use an acquisition function to investigate the most favorable areas of the resultant model. The goal function achieves a harmonious equilibrium between exploration and exploitation when determining new trial points. Bayesian optimization iteratively chooses a new point, assesses its performance using the objective function, and then updates the model. We repeat this procedure a certain amount of times to get the optimal value^35^.

### Performance evaluation

In this investigation, we used accuracy (acc), precision (pre), recall (rec), AUC, and Brier Score (bs) measures to assess the performance of the model. An accuracy metric is a quantitative evaluation that indicates the proportion of properly predicted samples by a model out of the total number of samples. A precision metric is a quantitative evaluation that indicates the ratio of correctly anticipated positive samples to the total number of expected positive samples. A recall metric precisely measures the ratio of correctly identified positive cases as positive. The AUC is a metric that quantifies the effectiveness of a classification model by calculating the area under the Receiver Operating Characteristic (ROC) curve. The ROC curve evaluates the algorithm’s ability to differentiate between various threshold values. The Brier score is a performance metric that quantifies the precision of probabilistic predictions. We calculate it by averaging the squared differences between the expected probability and the observed outcomes. A lower Brier score indicates that the model’s predictions align more closely with the actual outcomes. In addition to these criteria, we also computed net benefit ratings to provide a more accurate comparison of model performances. The Net Benefit Score is a quantitative measure that takes into account both the advantages of accurate positive forecasts and the disadvantages of incorrect positive predictions. We use this measure to evaluate the model’s clinical usefulness and efficacy at a specific threshold value. A high Net Benefit Score indicates that the model’s predictions are very valuable in the decision-making process and provide more favorable outcomes in real-world scenarios. The net benefit is crucial for assessing the practical efficacy of a model’s predictions, particularly in domains like medical diagnosis and treatment. This evaluation not only measures the model’s statistical accuracy but also its efficacy in clinical applications. We compute the Net Benefit Score by dividing the proportion of accurate positive forecasts to the entire population by the cost of inaccurate positive predictions. This computation evaluates the trade-off between the beneficial outcomes of the model and the negative consequences of false positives, based on a certain level of risk^36^.

### Morris sensitivity analysis

We use MSA, a global sensitivity analysis method, to evaluate the effects of model inputs on results. MSA examines each input variable’s impact on the model output separately and aims to determine the importance of these effects. With a one-at-a-time (OAT) design, MSA modifies only one input variable at a time, keeping all other variables constant. We repeat OAT at specific intervals for each input variable, recording the model output for each change step. The Morris method refers to the effects of these steps as elementary effects. We summarize the main effects by calculating the mean and standard deviation for each input variable. The mean value quantifies the overall impact of a variable on the model output, whereas the standard deviation reflects its level of stability or fluctuation. A large mean and small standard deviation imply a substantial and consistent impact, whereas a large standard deviation may reflect more intricate or interaction effects^25^.

### Proposed architecture

**Fig. 1:**
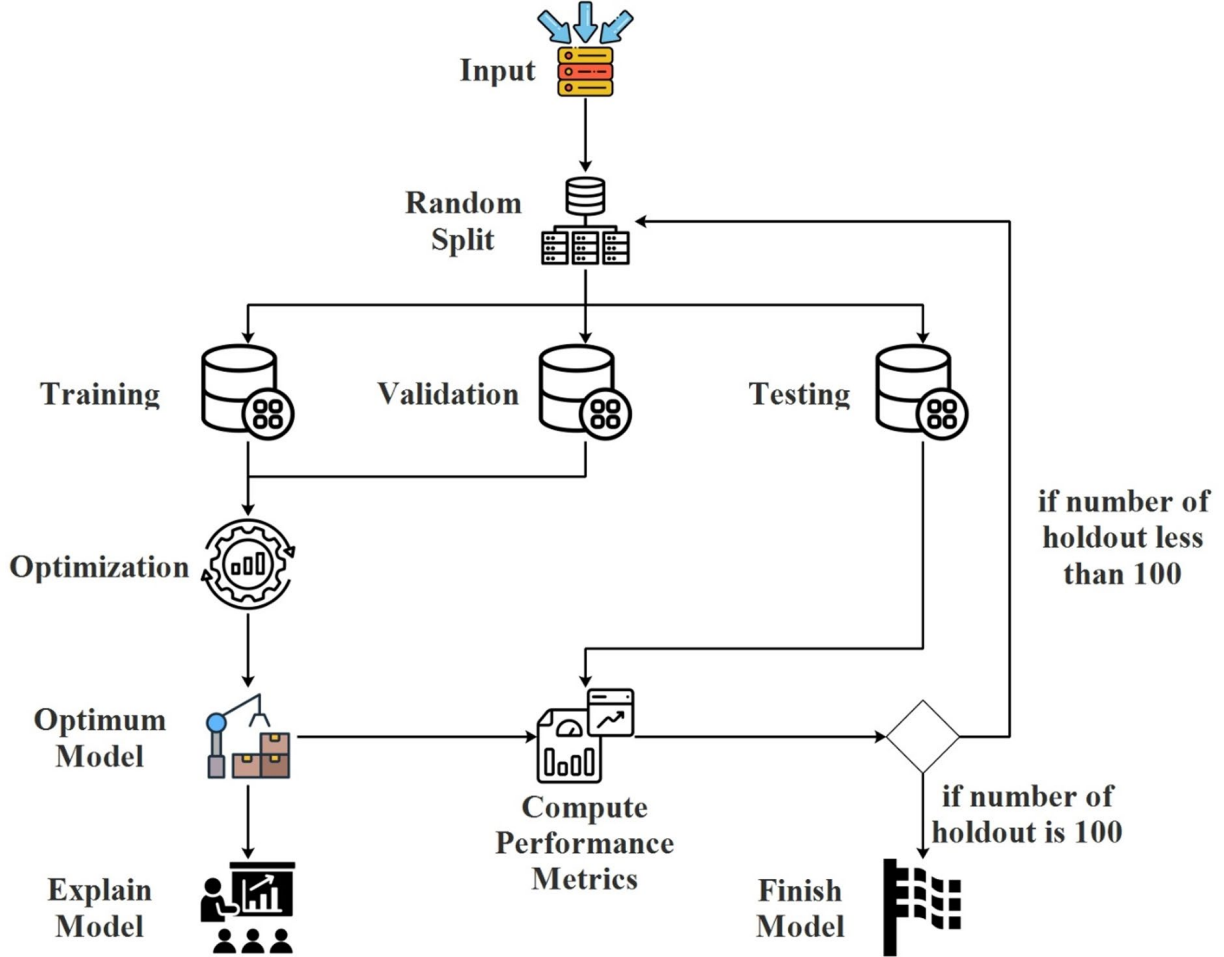
The schematic view of the proposed model process flow.

In the first step of the proposed model, the dataset was randomly divided into three to generate training, testing, and validation datasets. After this process the model was trained using a training dataset and the performance scores were computed using validation datasets. This stage was repeated a certain number of times to find the model that has optimal hyperparameter sets. The Bayesian optimization technique was used in the hyper-parameter optimization. At the end of this stage, the model that achieved the best metric score was determined, the performance scores of the model on the test data were calculated, and the model was explained using the MSA. After calculating the performance metric, if the holdout method was repeated 100 times, the proposed model was completed, otherwise, the random splitting stage was returned to repeat the holdout method. The process flow diagram of the proposed model is shown in Fig. 1.

## Results

The study population consisted of 430 control subjects and 425 patients with AHF. Significant demographic differences were observed between groups, with AHF patients being older (64.21 ± 9.96 vs. 58.35 ± 11.28 years, *p* < 0.001) and predominantly male (64.94% vs. 48.84%, *p* < 0.001). Comprehensive hematological analysis revealed distinct patterns in AHF patients compared to controls. The inflammatory profile was characterized by elevated white blood cell count [8.19 (2.79) vs. 7.49 (2.325) × 10⁹/L, *p* < 0.001] with increased neutrophil count [5.13 (2.5) vs. 4.295 (1.758) × 10⁹/L, *p* < 0.001] and decreased lymphocyte count [1.87 (1.13) vs. 2.29 (0.92) × 10⁹/L, *p* < 0.001], resulting in a significantly elevated neutrophil-to-lymphocyte ratio [2.7 (2.288) vs. 1.802 (0.941), *p* < 0.001]. Red blood cell parameters demonstrated evidence of anemia and altered erythropoiesis in AHF patients, including lower hemoglobin levels [13.2 (2.2) vs. 13.65 (2.1) g/dL, *p* < 0.001], reduced hematocrit [39.79% (7) vs. 40.9% (5.6), *p* < 0.001], and decreased mean corpuscular volume [9.69 (2.2) vs. 10.05 (1.3) fL, *p* < 0.001]. Additionally, increased red cell distribution width indices (RDW-SD and RDW-CV) suggested enhanced anisocytosis in the AHF cohort (both *p* < 0.001). Platelet parameters showed a statistically significant decrease in platelet count [241 (78) vs. 256 (84) × 10⁹/L, *p* < 0.001] with markedly increased platelet distribution width [16.39 (3.1) vs. 12 (3.475) fL, *p* < 0.001], indicating altered platelet morphology and heterogeneity in AHF patients.

These findings collectively demonstrate a complex hematological phenotype in AHF, characterized by systemic inflammation, impaired erythropoiesis, and altered platelet function, which may contribute to the pathophysiology and clinical outcomes in this population (Table 1).

**Table 1:** Statistical analysis of changes in blood parameters according to groups.

White blood cell parameters	Control group	AHF group	Statistical significance*
Total WBC count (×10⁹/L)	7.49 (2.33)	8.19 (2.79)	< 0.001
Neutrophil count (×10⁹/L)	4.295 (1.76)	5.13 (2.5)	< 0.001
Lymphocyte count (×10⁹/L)	2.29 (0.92)	1.87 (1.13)	< 0.001
Monocyte count (×10⁹/L)	0.56 (0.22)	0.64 (0.30)	< 0.001
Eosinophil count (×10⁹/L)	0.14 (0.13)	0.13 (0.15)	0.766
Basophil count (×10⁹/L)	0.04 (0.04)	0.03 (0.08)	0.041
Red blood cell and related parameters			
RBC count (×10¹²/L)	4.70 (0.68)	4.59 (0.75)	0.008
Hemoglobin (g/dL)	13.65 (2.10)	13.20 (2.20)	< 0.001
Hematocrit (%)	40.90 (5.60)	39.79 (7.00)	< 0.001
Mean corpuscular volume (fL)	10.05 (1.30)	9.69 (2.20)	< 0.001
Mean cell hemoglobin (pg)	29.10 (2.48)	28.80 (2.90)	0.029
MCHC (g/dL)	33.20 (2.00)	33.29 (1.90)	0.525
Red cell distribution and variability ındices			
RDW-SD	41.00 (4.80)	43.9 (6.40)	< 0.001
RDW-CV	13.20 (1.30)	14.60 (2.69)	< 0.001
Platelet parameters			
Platelet count (×10⁹/L)	256.00 (84.00)	241.00 (78.00)	< 0.001
Platelet distribution width (fL)	12.00 (3.48)	16.39 (3.10)	< 0.001
Procalcitonin (%)	0.25 (0.07)	0.22 (0.08)	< 0.001
Calculated ratios			
Neutrophil/Lymphocyte ratio	1.80 (0.94)	2.70 (2.29)	< 0.001

*: Descriptive statistics are presented as median (IQR) and statistical hypotheses were performed according to the Mann-Whitney U test.

In the second stage of the study, predictive models were constructed with ML approaches using hematological parameters. The dataset was divided into three parts to generate training, testing, and validation datasets. For this purpose, 10% of all samples were randomly selected to generate the testing dataset, 10% were randomly selected to generate the validation dataset, and the remaining samples were used to generate the training dataset. For dataset splitting, the train_test_split function from the sklearn library was used with the stratify parameter, ensuring that the class distribution was balanced across the training, testing, and validation datasets. As mentioned in the proposed model, this process was repeated 100 times. Each time, the hyper-parameters of each model were optimized, and performance scores were computed. The Bayesian optimization method was developed using scikit-optimize library of Python to optimize hyperparameter of models. From this library, gp_minimize function was used with parameters acq_func=’EI’ and n_calls = 50. For model optimization, the accuracy obtained in the validation dataset was maximized. The hyperparameter names and parameter spaces optimized for each model are shown in Table 2. This table does not include the optimum hyperparameters for each model, as each model has 100 optimum hyperparameter spaces. While the values in this table can be integers or real numbers and take any value within the specified range, the categorical values can only take values from the elements in the list (Table 2).

**Table 2:** Hyper-Parameters Name, type and space for bayesian optimization Method.

Model name	Hyper-parameter name	Hyper-parameter type	Hyper-parameter space
EBM	max_bins	Integer	Low: 2^9^, High:2^20^
	max_interaction_bins	Integer	Low: 2^2^, High:2^8^
	Interactions	Categorical	[0.25, 0.5, 0.75, 0.95, 5, 10, 25, 50, 100, 250]
	outer_bags	Integer	Low: 10, High: 75
	inner_bags	Integer	Low: 0, High: 10
	learning_rate	Real	Low: 10^−4^, High: 10^−1^
	greedy_ratio	Categorical	[0.0, 0.25, 0.5, 0.75, 1.0, 1.25, 1.5, 1.75, 2.0, 4.0]
	min_samples_leaf	Categorical	[2, 3, 4, 5, 6]
	min_hessian	Categorical	[0.1, 0.01, 0.001, 0.0001, 0.00001, 0.000001]
	max_leaves	Categorical	[3, 4, 5, 6, 7, 8, 9]
ETC	n_estimators	Integer	Low: 50, High: 500
	Criterion	Categorical	[“gini”, “entropy”, “log_loss”]
	min_samples_split	Integer	Low: 2, High: 20
	min_samples_leaf	Integer	Low: 1, High: 20
histGB	max_iter	Integer	Low: 50, High: 1000
	max_leaf_nodes	Integer	Low: 2, High: 50
	min_samples_leaf	Integer	Low: 2, High: 50
	l2_regularization	Real	Low: 0, High: 0.9
	Tol	Real	Low: 10^−10^, High: 10^−5^
KTBoost	learning_rate	Real	Low: 0.001, High: 0.3
	max_depth	Integer	Low: 2, High: 10
	min_samples_leaf	Integer	Low: 1, High: 10
LR	max_iter	Integer	Low: 50, High: 5000
	Tol	Real	Low: 10^−6^, High: 10^−2^
	C	Real	Low: 2^−11^, High:2^11^
MLP	hidden_layer_sizes	Integer	Low: 50, High: 1500
	Activation	Categorical	[“identity”, “logistic”, “tanh”, “relu”]
	learning_rate_init	Real	Low: 10^−8^, High: 10^−3^
	max_iter	Integer	Low: 100, High: 400
	Alpha	Real	Low: 10^−9^, High: 10^−3^
RF	n_estimators	Integer	Low: 50, High: 500
	Criterion	Categorical	[“gini”, “entropy”, “log_loss”]
	min_samples_split	Integer	Low: 2, High: 20
	min_samples_leaf	Integer	Low: 1, High: 20
XGBoost	learning_rate	Real	Low: 0. 00001, High: 0.01
	min_split_loss	Integer	Low: 0, High: 100
	max_depth	Integer	Low: 4, High: 30
	reg_lambda	Integer	Low: 0, High: 30
	reg_alpha	Integer	Low: 0, High: 30

We optimized the hyper-parameters of each model proposed in the study separately for each holdout using the values shown in Table 1. In Table 2, the hyperparameter ‘Interactions’ for EBM does not represent categorical variables per se but rather specifies the maximum number of pairwise interaction terms to consider during model training. The values correspond to discrete options provided by the ‘interpret’ library, which internally manages these as selectable settings for interaction depth and ratio. We determined the optimal hyperparameters based on the accuracy of the validation dataset. We developed all classification models using the Python programming language. We used the interpret library for EBM among these models. Following the hyperparameter optimization phase, we calculated the performance scores of the trained model using the optimal hyperparameters on the test dataset. Table 3 shows the acc, pre, rec, AUC, and bs values calculated for each model. These scores, shown in Table 3, are the averages across all holdouts for each model. Therefore, Table 3 also provides the standard deviation (SD) values for each metric, calculated using the scores across holdouts (Table 3).

**Table 3:** Holdout mean performance score of machine learning models on testing dataset.

Model Name	acc (%)	sd_acc	pre (%)	sd_pre	rec (%)	sd_rec	AUC (%)	sd_ AUC	bs	sd_bs
EBM	87.26	2.01	88.31	3.88	86.23	4.51	87.58	2.84	0.12	0.02
ETC	86.79	1.39	87.46	3.45	86.23	4.26	86.91	2.77	0.12	0.01
histGB	87.40	1.96	88.59	2.24	85.95	4.44	87.93	2.47	0.11	0.02
KTBoost	85.77	1.10	87.97	2.56	83.07	3.75	85.12	2.21	0.13	0.01
LR	87.58	1.46	88.16	3.71	87.16	3.95	87.33	2.95	0.11	0.01
MLP	84.70	1.82	83.53	3.29	86.79	4.59	84.62	3.65	0.14	0.02
RF	87.67	1.32	88.69	3.37	86.70	4.34	87.55	2.71	0.11	0.01
XGBoost	84.19	1.23	87.83	3.56	79.72	4.58	83.71	2.32	0.14	0.01

**Fig. 2:**
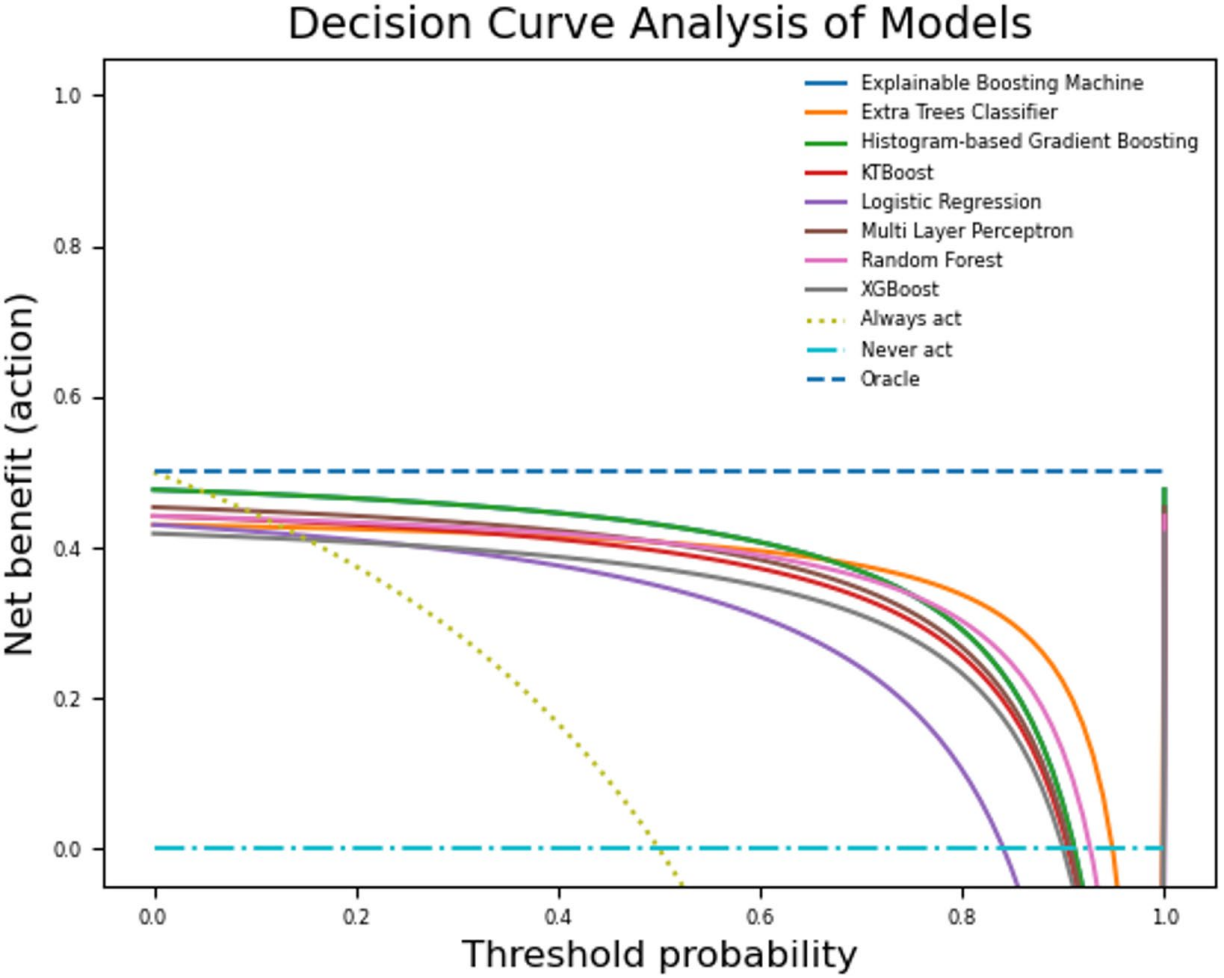
Net benefit display of each model that obtained the best scores across holdouts.

Examining the values in Table 3, we observe that the RF algorithm achieves the highest accuracy. However, in addition to the RF algorithm, the EBM, histGB, and LR algorithms also achieve an accuracy better than 87%. The RF, LR, and histGB methods achieve the best results in the precision, recall, and AUC metrics, respectively. In terms of the Brier score, the results are similar, but the histGB, LR, and RF algorithms outperform the other methods. AUC was selected as the primary evaluation criterion in the study, and when the AUCs were examined, the histGB model (87.93%) obtained a higher AUC value than all other models. To make a better comparison between models, it is crucial to evaluate the algorithms using different metrics. In this context, we recorded the standout model that achieved the best score for each classification algorithm and drew a net benefit graph using these models. Figure 2 shows the net benefit graph drawn for the best holdout scores of the models. This graph was created using the statkit library available in Python.

Although RF achieved the highest accuracy, we selected AUC as the primary model selection criterion. AUC was chosen because it is threshold-independent and more robust for evaluating models in imbalanced medical datasets, where sensitivity and specificity trade-offs are clinically critical. Based on AUC, histGB was identified as the best-performing model (AUC = 87.93%). To investigate whether the model exhibited differential performance across sexes, an additional analysis was conducted. The test set instances were stratified by sex, and AUC values were computed separately for female and male groups. The histGB model, identified as the best-performing model, was employed for this analysis. The results indicated that the histGB model achieved an AUC score of 87.7 for female participants and 88.2 for male participants. As the cohort was ethnically homogeneous (all Turkish), race/ethnicity-based stratification was not applicable.

**Fig. 3:**
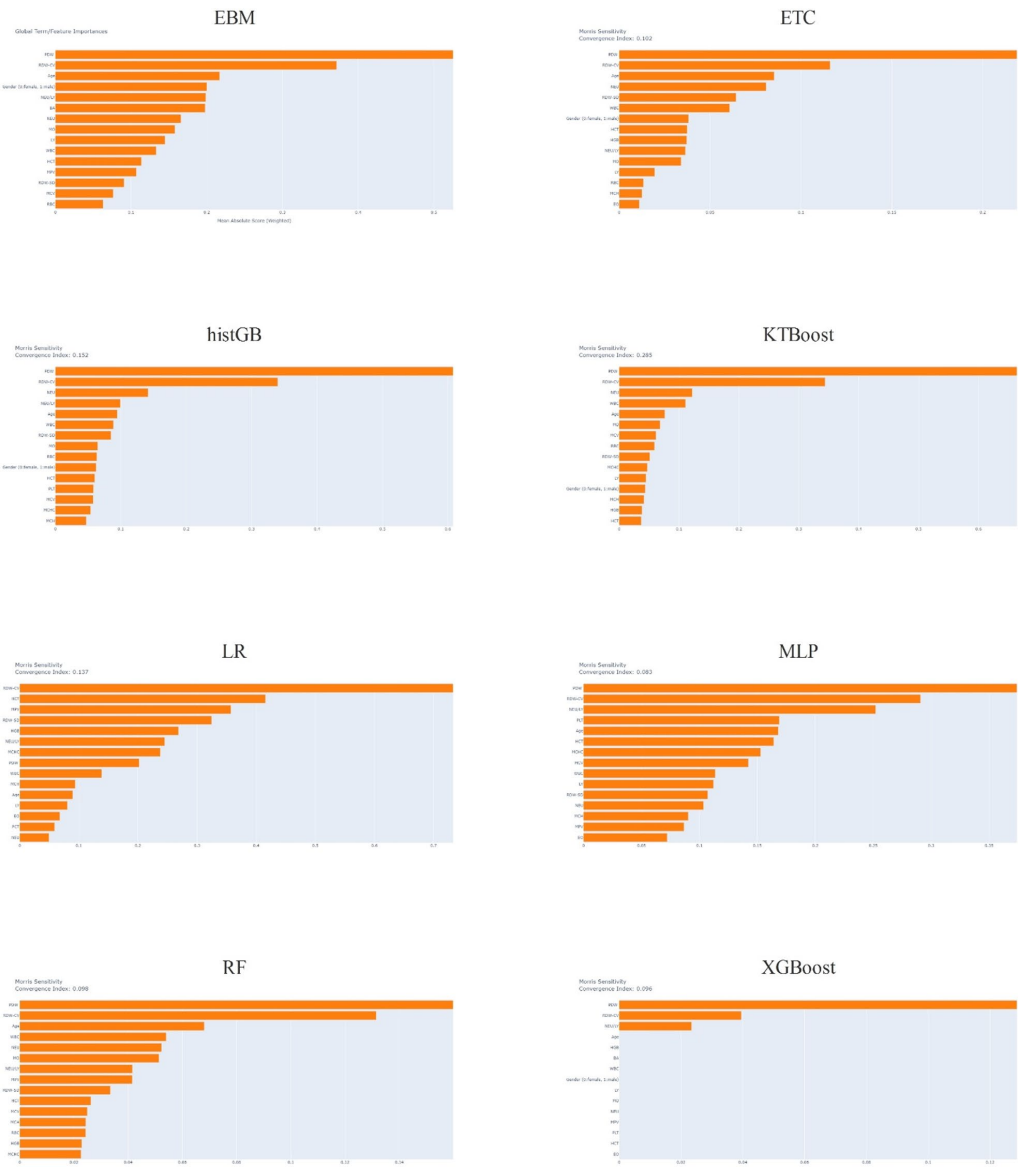
Model explanation graph for all ML algorithms.

In this chart, the line marked “Oracle” represents the perfect model, while the line marked “Never act” represents the worst model. In this context, we observe that the ETC and RF models outperform other models in terms of net benefit scores. We observe that models other than LR, ETC, and RF produce a similar graph. The features used in the dataset are one of the most important factors affecting machine learning models’ performance. Measuring feature effects, especially in disease diagnoses, is critical to understanding which variable plays a greater role in detection. We used the Morris method to explain the model that achieved the highest score among the holdouts for each ML method in our study. Figure 3 shows the graphics obtained from the explanation for each model.

## Discussion

The current research makes a noteworthy addition to the field by combining XAI with hematological biomarkers to provide a new diagnostic approach for AHF. The results of this study have the potential to optimize healthcare processes and improve patient outcomes. Using XAI’s capabilities, the examination of hematological markers provides fresh perspectives on the prompt and precise identification of AHF, possibly revolutionizing healthcare provision. The present study discovered that platelet distribution width (PDW) and red blood cell distribution width (RDW-CV) were the two most important factors in predicting AHF using various machine learning models. However, in the case of logistic regression (LR), the most predictive factors were RDW-CV and hem-atocrit (HCT). Age, PDW, RDW-CV, neutrophil count (NEU), lymphocyte count (LY), and the NEU/LY ratio all have significant values after a thorough analysis of the top five characteristics across all models. The findings emphasize the critical role of hematological indicators in forecasting AHF, which may serve as a useful instrument for prompt detection. Furthermore, the research shows that all machine learning models used attained an accuracy rate of 84%, indicating the significant potential of these methods in diagnosing AHF. We found the histogram-based gradient boosting model to be the most successful, achieving an AUC value of 87.93%. The MSA and the EBM consistently ranked PDW, RDW-CV, NEU, NEU/LY ratio, age, and WBC as some of the most important factors, appearing in the top 10 in at least six models. Putting together MSA (model-agnostic supervisory algorithm) and XAI makes a framework that is simple to understand and makes machine learning models more useful in clinical settings. XAI enhances the comprehensibility of the decision-making process, allowing healthcare practitioners to have more confidence in and make better use of these models in real-world situations. The MSA technique’s unique early detection of AHF, previously unreported in the literature, underscores the significance of these results. This study establishes the foundation for future research and clinical applications, providing a hopeful direction for improved identification and therapy of AHF.

The MSA complemented similar findings on several other markers, including PDW, RDW-CV, and the NLR which were found to have a high level of stability across different machine-learning models. This gives a strong feature ranking which is in line with past literature recommending several de novo inflammatory and hematological biomarkers be considered highly effective for the pathophysiology of AHF^37,38^. Thus, this investigation offers a new application of explainable deep learning concepts, which might be useful in improving patient outcomes by making model-based clinical decisions across healthcare systems to identify patients with higher risk of mortality.

The decision curve analysis (Fig. 2) demonstrated that RF and ETC provided higher net benefit at clinically relevant threshold probabilities (10–30%). In practice, this implies that adopting these models for decision-making could lead to improved identification of true-positive AHF patients without substantially increasing unnecessary interventions. Thus, the net benefit analysis highlights the potential clinical utility of our models in supporting early diagnostic strategies.

PDW is a superior measure of platelet activation in comparison to mean platelet volume since it stays constant throughout basic platelet swelling^39,40^. Growth factors and cytokines present in atherosclerotic processes may cause an increase in PDW levels by inhibiting platelet production in the bone marrow^41,42^. Platelets that are larger exhibit higher levels of metabolic and enzymatic activity compared to smaller platelets^43^. Activated platelets initiate the process of blood clot formation, which contributes to the development of atherothrombotic illness when there is a rupture in the plaque that builds up in the arteries or damage to the cells lining the blood vessels^44^. Regarding prog-nosis, elevated levels of PDW are a separate predictor of cardiac death and negative cardiovascular events in individuals with coronary artery disease (CAD)^39,45^. A prospective observational study of 1,746 patients hospitalized for HF reported that a high PDW value was a predictor of poor prognosis in patients with HF^46^. A medical paper examined the average size of platelets, the number of platelets, and the estimation of platelet size distribution in 59 patients with diabetes and 88 patients without diabetes who had experienced a heart attack. They compared these results with those of 100 individuals without diabetes and 50 patients with diabetes who did not have a heart attack. The authors stated that irregularities in platelet function could potentially contribute to the comparatively unfavorable prognosis of myocardial infarction in diabetes patients^47^.

Certain cardiovascular illnesses regard elevated RDW as a risk factor. Studies have linked medical conditions such as heart failure, coronary artery disease, atrial fibrillation, and an increased risk of death associated with cardiovascular issues to an elevated Red Cell Distribution Width (RDW)^48,49^. High RDW levels could mean that red blood cells are less stable and less effective, which could be a sign of deeper problems like chronic inflammation, oxidative stress, or not getting enough nutrients. The RDW-CV is an RDW version with increased sensitivity. A high RDW-CV value suggests significant variability in the size of red blood cells, which could potentially be associated with cardiovascular risks. Hence, in this research, we examined the use of RDW-CV as a biomarker for AHF. The RDW-CV biomarker showed a high level of significance in our XAI model. It should be noted that identifying PDW and RDW-CV as the most significant hematological biomarkers, which might help the prognosis of essential AHF, is of particular concern. Previous works have already provided concrete evidence pointing out the fact that high levels of PDW are predictors of a worsened prognosis in heart failure and other types of cardiovascular diseases^46^. Likewise, elevated RDW-CV was found in addition to poor cardiovascular prognosis and raised mortality^48,49^. These study findings endorse the usefulness of conventional hematological parameters for risk assessment in AHF patients. They can serve as a routine laboratory marker that has low cost and is widely available. Adding the biomarkers into the XAI model gives the clinician an objective way of pinpointing high-risk patients in the initial management setting and may warrant extra targeted interventions.

Both advanced ischemic and nonischemic heart failure are associated with neutrophils and monocytes/macrophages. In AHF, hyperactive inflammation is caused by a buildup of neutrophils and their prolonged activation. In chronic heart failure, this inflammation affects the prognosis over time. Following a myocardial infarction, the first cohort of inherently reactive and transient neutrophils plays a crucial role in instigating the inflammatory response, reducing inflammation, and restoring the heart. Nevertheless, if neutrophils are overly and consistently stimulated, they have the potential to inflict more harm on the heart tissue. A research study discovered a significant correlation between an elevated neutrophil count at admission and the rapid development of congestive heart failure^50^. According to a paper^51^, neutrophils play a crucial role in promoting heart repair after myocardial infarction by guiding macrophages toward a regenerative state. Researchers discovered that neutrophils play a big role in ischemic cardiomyopathy and could be used to target the immune system in order to treat this illness^52^.

NLR can be easily and affordably determined by a standard blood test for patients who are admitted to the hospital. Cho et al.^37^ found that a high NLR upon admission to the hospital in patients with AHF was a significant predictor of both in-hospital mortality and mortality within three years following release. A study^38^ found that the NLR could be valuable in categorizing the risk of patients admitted to the hospital with acute heart failure and preserved ejection fraction (HFpEF).

Pieszko et al.^53^ used an ML model to predict in-hospital mortality in patients with acute coronary syndromes. The authors reported that they achieved 81% sensitivity and 81.1% specificity in predicting in-hospital mortality with a dominance-based rough set approach and a model based on the full set of laboratory and clinical features. Adler et al.^54^ trained a decision tree algorithm to associate a subset of patient data with very high or very low mortality risk in a cohort of 5822 hospitalized and outpatient patients with HF. The authors reported a risk score that accurately discriminated between low and high mortality risk using markers such as diastolic blood pressure, creatinine, hemoglobin, white blood cell count, platelets, and red blood cell distribution width and the model had an AUC of 0.88. Recent advances in AI-driven cardiovascular diagnostics have emphasized the critical importance of addressing algorithmic bias and ensuring model transparency across diverse patient populations. Contemporary studies employing advanced signal processing techniques, such as FBSE-based feature extraction combined with ensemble learning approaches, have demonstrated exceptional performance in cardiac arrhythmia detection, achieving accuracies exceeding 99% while maintaining computational efficiency suitable for wearable devices^55^. Similarly, the integration of fuzzy logic systems with deep neural networks for ECG-based cardiac classification has shown superior performance in handling data uncertainty and improving interpretability, particularly in distinguishing minority arrhythmia classes^56^.

The results indicated that the histGB model achieved the largest AUC of 87.93%, establishing it as the best performer. It is worth mentioning that other models, namely RF and EBM, also performed well with an accuracy of over 87%. However, they did not outperform histGB in terms of AUC. This discrepancy highlights the significance of using several assessment criteria in evaluating the practical application of models, as accuracy alone may not provide a comprehensive understanding of the effectiveness of models in identifying AHF.

The majority of the aforementioned research focused primarily on the analysis of hematological parameters and cardiac issues using statistical methodologies. Several studies have constructed machine learning models; however, they have not adequately elucidated the opaque character of these models. MSA helped to clarify the intricate models used in this study. The study’s results showed that combining machine learning with MSA effectively discovered significant hematological indicators for categorizing AHF. The current inquiry is subject to many limits. We conducted the research at a single center without external validation. However, for the sample computation, the sample size was rather large, and we used a robust validation mechanism for the machine learning models. In addition, this research did not use omic data, such as genomic, proteomic, or metabolomics data. Instead, its main emphasis was on exploring the influence of hematological markers in identifying AHF. Additional research is required to develop models that can accurately forecast AHF using both omic-level data and hematological indicators. Using the machine learning approach, we have developed a classification model that can accurately distinguish individuals with AHF. The model that used readily available hematological variables produced consistent results. These results suggest that the model has the potential to be a significant tool in assisting the treatment of persons with AHF. The Complete Blood Count is a very useful method for promptly identifying and diagnosing medical conditions in clinical environments, thanks to its straightfor-wardness and cost-effectiveness. Furthermore, this research’s complete methodology has the potential to aid in evaluating the probability of events in various medical situations.

In sum, this work presents an integrated methodology that incorporates XAI with MSA to propose a new and understandable diagnostic framework for AHF. Hematological biomarkers’ application resulted in the creation of cost-effective and easily accessible machine learning models to diagnose the disease and develop a custom treatment. The proposed approach of integrating explainable AI is aimed at increasing the clinical relevance of AI systems and, thus, the chance to apply them in practice and to affect the patients’ experiences.

## Data Availability

The raw data supporting the conclusions of this article will be made available by the authors on request.
